# Paternal age at conception effects on offspring telomere length across species—What explains the variability?

**DOI:** 10.1371/journal.pgen.1007946

**Published:** 2019-02-14

**Authors:** Dan T. A. Eisenberg

**Affiliations:** 1 Department of Anthropology, University of Washington, Seattle, Washington, United States of America; 2 Center for Studies in Demography and Ecology, University of Washington, Seattle, Washington, United States of America; The University of North Carolina at Chapel Hill, UNITED STATES

Telomeres, the repeating DNA sequences found at the ends of chromosomes, are dynamic and complex parts of the genome with important health and fitness consequences. In this issue of *PLOS Genetics*, Bauch and colleagues [1] help to extend our understanding of telomere biology in important ways. Although telomeres are DNA, they do not act like DNA is “supposed” to. Telomeres get shorter with each round of mitosis due to the end replication problem and in response to DNA damage. They can be extended by telomerase, among other mechanisms. This means that telomere length (TL) may change over an organism’s life (as soon as a fertilized egg begins dividing) and do so differentially across cells in the body, reflecting the particular replicative and telomere maintenance mechanisms experienced by a cell lineage.

Growing evidence suggests that gametes are not exempt from these complex telomere dynamics and that TL does not adhere to canonical DNA inheritance patterns. First, the telomeres contained on particular chromosome ends within an egg and sperm cell are the telomeres that a fertilized egg begins its life with [2]. Conventional genetic polymorphisms have been shown to predict TL in humans. These genetic polymorphisms could influence TL via effects on TL dynamics from the first mitotic division of the fertilized egg on. However, genetic polymorphisms could also predict TL via indirect genetic effects on telomere dynamics in the mother or father’s gamete cell lineages, which are then passed on to offspring. Although the gametes of female animals are generally produced before birth, males must continually produce sperm. Consistent with this, paternally transmitted TL appears to be dynamic, whereas maternally transmitted TL is less so. In particular, paternal age at conception (PAC) seems to influence sperm TL, and subsequently the TL of offspring, whereas maternal age at conception (MAC) does not [3].

Further complicating the picture, telomere dynamics vary considerably across species (Table 1). In cross-sectional studies of humans and chimpanzees, sperm TL and, correspondingly, offspring TL appears to increase with PAC. Other species have tended to show either no association between PAC and offspring TL, or a negative association.

**Table 1:** Paternal age effect on offspring TL across species (adapted and updated from [3]).

Species	n	r^a^	p	Longevity^b^^,^^c^	Weight (g)^b^	Reference
Atlantic salmon	60		NS	13	25,740	[12]
Sand lizard*	12	-0.59	0.041	20	15	[13]
European shag	204	+	0.43	30.6	1,773	[14]
Common tern*	142	−	0.02	33	120	[5]
Alpine swift*	95	−	0.033	26	102.7	[15]
Zebra finch*	139	−	0.032	12	12	[16]
Jackdaw*	715	−	0.007	20.3	246	[1]
Great reed warbler	154	+	0.7	10.1	30	[17]
Soay sheep	318	0.066	0.238	22.8	80,000	[18]^d^
House mouse*	12^d^	−	≤0.05	4	20.5	[19]
Long-tailed macaque^e^	9	+	NS	39	6,363	[20]
Chimpanzee*	40	0.42	0.009	59.4	44,984	[4]
Human*	144	0.15	0.03	122.5	62,035	[4]

*p < 0.05

^a^correlation values if reported; otherwise “+” indicates positive association and “−” negative association

^b^from AnAge database except sand lizard data which came from https://www.wildlifetrusts.org/wildlife-explorer/reptiles/sand-lizard

^c^maximum longevity

^d^personal communication

^e^testicular TL instead of offspring TL

**Abbreviations:** n, number of offspring; NS, non-significant; p, p-value; r, correlation coefficient; TL, telomere length.

Bauch and colleagues’ study [1] extends the literature on parental age and offspring TL in particularly robust ways. They take advantage of a long-term study of jackdaw birds to marshal a large, longitudinal and family-based sample. TL was carefully measured using telomere restriction fragment (TRF) analysis and met high–quality-control standards. Additionally, a cross-fostering experiment was also conducted. Because of these combined strengths, the results allow better causal inferences than is typical in studies of TL inheritance patterns.

Although converging evidence strongly suggests that the PAC association with offspring TL in humans is caused by a progressive increase in sperm TL as men age [3], most studies in humans and other species have been cross-sectional and observational. Therefore, doubt often remains about the degree to which PAC associations represent changes in gamete TL versus other causal pathways. For example, TL may predict longevity and health such that males who start life with longer TL tend to live to a later age and transmit their own longer telomeres to offspring. Such a dynamic could create a correlation between PAC and TL without requiring a lengthening of sperm TL with age.

By comparing siblings sired by the same father at different PACs, Bauch and colleagues [1] showed clear evidence that TLs of offspring are likely shortened as fathers age. Cross-fostering, buttressed these findings by showing that biological, but not foster-father, ages predicted offspring TL. Similar analyses showed no apparent effect of MAC on offspring TL. Although it is possible that other factors confound this analysis, such as mothers adjusting egg contents based on the age of their mates, this study provides strong evidence that telomeres are shortened with paternal age in this species.

It remains unclear what the mechanistic and evolutionary explanations for the PAC effects on TL are, and why the effect varies across species. One explanation for cross-species variability that has garnered some support is that species with higher sperm production rates show a greater increase in sperm TL with age [3–5]. Surveying the most recent evidence of PAC associations with TL across species (Table 1), an alternative possible explanation emerges. It appears that increases in TL with PAC are generally found more in longer-lived and larger species, whereas shorter-lived and smaller species generally show negative PAC effects or no effect at all. Past studies have shown that long-lived species generally have shorter TL, whereas larger species generally have lower somatic telomerase activity (TA) [6, 7]. Lower somatic TA in large organisms is thought to be driven by the greater risk of cancer development in these species, which selects against the cancer promoting effects of TA. Therefore, the diversity of these PAC effects on TL is all the more puzzling because the most prominent suggested cause of a positive PAC effect is high expression of testicular TA. If larger body size increases TA-promoted cancers in both somatic and germ line tissues, the expectation is that long-lived species should have decreased testicular TA and more negative PAC effects on TL, not more positive ones. Examination of testicular TA levels across species may help better reveal the extent to which testicular TA levels underlie cross-species variation in PAC effects.

This contradiction between expected testicular TA levels and PAC effects on TL suggest that explanations other than testicular TA should also be further explored. There is evidence that changes in sperm TL in humans are, at least partly, driven by selective survival or proliferation of spermatogonial stem cells with longer TL [3, 8]. Given this, future studies of the PAC effect in humans and other species should consider not just changes in mean TL with PAC, but changes in TL distributions. For example, the TRF analysis deployed by Bauch and colleagues provides data on the distribution of TL, which could be analyzed in future studies to help address whether there are shifts not only in the TL mean, but also the TL distribution with PAC.

Regardless of mechanisms, the PAC effect on TL has intriguing health and evolutionary implications. Strong converging evidence suggests that TL influences health and longevity. The basic inheritance patterns of DNA as well as empirical evidence in humans suggests that the PAC effect persists across generations [9]. Therefore, if ages of reproduction change in a lineage/population in which PAC effects exist, then the TL of descendants could be rapidly and durably shifted in ways that change their health and fitness. This raises the question of why PAC effects persist if they cause such phenotypic instability. I have suggested that the positive PAC effect in humans might represent a unique type of adaptive intergenerational genetic plasticity wherein descendants’ TL are progressively adjusted based on average age of reproduction among male ancestors [10]. If ages of reproduction of ancestors are predictive of the environment descendants will experience, such a mechanism could allow a more appropriate TL for that environment. Specifically, in lineages with later ages of reproduction, longer TL may promote increased maintenance effort that improves fitness in the context of low extrinsic mortality environments. How do we square this with the emerging evidence from Bauch and colleagues as well as others (Table 1) that the PAC effect on TL is usually nonexistent or negative in smaller and shorter-lived species? Adaptive intergenerational effects are more likely to emerge when intragenerational plasticity is constrained [11]. Because larger species tend to have low somatic TA, this might constrain their intragenerational plasticity in TL and thereby increase selection for intergenerational plasticity.
